# Predictors of Baboon Sleep Site Selection in Gorongosa National Park

**DOI:** 10.1002/ajpa.70095

**Published:** 2025-08-01

**Authors:** Lynn Lewis‐Bevan, Philippa Hammond, Susana Carvalho, Dora Biro

**Affiliations:** ^1^ Behaviour and Biomechanics, Department of Biology University of Oxford Oxford UK; ^2^ Primate Models for Behavioural Evolution Lab, School of Anthropology and Museum Ethnography University of Oxford Oxford UK; ^3^ The Interdisciplinary Centre for Archaeology and the Evolution of Human Behaviour Universidade do Algarve – Campus de Gambelas Faro Portugal; ^4^ Department of Science Gorongosa National Park Gorongosa Sofala Mozambique; ^5^ CIBIO/InBIO, Centro de Investigação em Biodiversidade e Recursos Genéticos Universidade do Porto Vairão Portugal; ^6^ Department of Brain and Cognitive Sciences University of Rochester Rochester New York USA

**Keywords:** baboon behavior, sleep site location, sleep site selection

## Abstract

**Objectives:**

This research aimed to understand how sleep site selection compared to other study sites in baboons living in a low‐predator density, highly seasonal environment. We compared baboon troops in two distinct habitat types with different seasonal influences within the park, one that flooded annually and one that did not. We compared their sleep site use, reuse, and location relative to home range boundaries and areas of interest (AOIs) with each other and baboons in other areas to understand whether season, habitat familiarity, or position in the home range influenced sleep site choice.

**Methods:**

Using GPS collar data taken at 15‐min intervals from four gray‐footed chacma baboons ( *Papio ursinus griseipes* ) in Gorongosa National Park, Mozambique, we established the location of sleep sites, home range boundaries, and AOIs, or places where the baboons repeatedly stopped for more than 15 min. Study subjects ranged either in dense woodland or in a seasonally flooded alluvial floodplain. We used a linear mixed‐effects model to predict sleep site reuse based on distance to the habitat edge and AOIs, and Wilcoxon signed‐rank tests to determine if morning or evening AOIs influenced sleep site location. We counted the number of reuses of each sleep site before and after the flooding period and compared this data to data in other baboon study sites.

**Results:**

We found that, as in other study sites with less seasonality and higher predation risk, baboons in Gorongosa change sleep site frequently and utilize multiple sleep sites throughout their home range, although they more often use sleep sites closer to the center of their home ranges. However, unlike other studies, we found that the location of the last AOI of the day more strongly predicted sleep site location than the first AOI of the next day in one troop, with baboons traveling further from their sleep site to their first AOI in the morning than from their last evening AOI to the sleep site.

**Conclusions:**

Despite high seasonality and low predator density, baboons in Gorongosa National Park changed sleep sites frequently, as do other studied baboon troops in areas with high nocturnal predation rates. In addition, their propensity to sleep closer to the last AOI of the day may imply that they plan their daily paths toward their chosen sleep site, or that they sleep opportunistically at the end of the day. This study provides a baseline of behavioral data for comparison to other sites and future work in Gorongosa, where predator density continues to rise since the time of the study.

## 1 Introduction

Sleep has vital functions in living creatures, contributing to homeostasis, attentiveness, brain development, and long‐term health (Cirelli and Tononi 2008). Choosing a suitable sleep site is essential for predation avoidance, socializing, and access to food (Altmann 1974; Burger et al. 2020; Butler and Roper 1996; Caselli et al. 2014; Chapman 1989; Savagian and Fernandez‐duque 2017). Sleep site selection is particularly important for the short‐term survival and long‐term fitness of individuals (Anderson 1984). Where an animal or animal group sleeps can affect predation risk (Chu et al. 2018; Ellison et al. 2019; Feilen and Marshall 2014; Gazagne et al. 2020; José‐Domínguez et al. 2015; Ogawa et al. 2014), thermoregulation (Chu et al. 2018; Ellison et al. 2019; Koops et al. 2012), proximity to resources such as food or water (Brividoro et al. 2019; Gazagne et al. 2020; Ogawa et al. 2014; Teichroeb et al. 2012), pathogen avoidance (Brividoro et al. 2019; Feilen and Marshall 2014), or conspecific avoidance and range defense (Brividoro et al. 2019; Markham et al. 2016).

Traditionally in primates, the study of sleeping patterns has been restricted to behavioral follows or captive primate studies, but the rise of biologging devices, such as those incorporating GPS loggers, has made it feasible to study the behaviors of cryptic or difficult‐to‐follow primates, as well as nocturnal behaviors where visual contact may be limited (Ayers et al. 2020; Campera et al. 2022). While handheld GPS devices have been used to track the location of sleeping sites in primates (e.g., Chu et al. 2018; Ellison et al. 2019), studies that equip animals with GPS collars to remotely record the sleeping patterns of wild primates have come about relatively recently. This rise of smaller, more accessible biologgers and improved quantitative treatments has made it easier for studies to collect and interpret large volumes of data.

The rise in the use of biologgers has caused an increase in the amount of available movement data within the primate clade (Dore et al. 2020). With some GPS trackers able to log data at sub‐second temporal resolution and high spatial precision, scientists can now identify individual decision points in a daily path, pinpoint key resources that may not be apparent to human observers, and look at group cohesion and leadership empirically in situations beyond human access (Byrne et al. 2009; Farine et al. 2017; Strandburg‐Peshkin et al. 2015). However, with the increase in data comes a need to test new forms of analysis, including on data where direct observational data were not available (Alavi et al. 2022; Janmaat et al. 2021; Kays et al. 2015).

One common approach to studying movement data without observation is to use remote sensing or environmental data, collected either from satellite imagery, monitors, or ground truthing. Among difficult‐to‐observe nocturnal primates, a review of Lorisiformes found that sleep site type and selection vary between sleep sites, and daytime predation played a role in sleep site selection (Svensson et al. 2018). Similarly, nocturnal lesser galagoes ( *Galago senegalensis* ) slept in areas with more forest cover and connectivity, in places with lower mean temperature, suggesting thermoregulation and predation risk influence their sleep site choice (Ellison et al. 2019).

Amongst the more easily observed diurnal primates, proboscis monkeys ( *Nasalis larvatus* ) picked sleeping trees that minimized molestation by insects and predation (Feilen and Marshall 2014), while howler monkeys ( *Alouatta caraya* ) also moved sleep sites to avoid parasites, as well as to be close to their first morning feeding site (Brividoro et al. 2019).

The sleeping patterns of the genus *Papio* have been extensively studied, in part due to their large body size, making the placement of biologgers more viable and the number of widespread, long‐term field sites. A group‐living, primarily terrestrial genus, *Papio* species are known to sleep on cliff faces, in trees, or in other elevated areas, choosing sites that provide safety from their primary nocturnal predator, leopards (Altmann and Altmann 1970, pp84‐85). These sites are often contested resources between baboon troops, with sites occurring in overlapping home range areas and used by multiple troops on consecutive nights (Altmann and Altmann 1970, p77; Markham et al. 2016).

Understanding why baboon troops use the sleep sites they do, and when they use them, is a common topic of study in the *Papio* genus. Suire et al. (2021) studied sleep site use in Anubis baboons ( *Papio anubis* ) in relation to rainfall and resource availability, and found that while sleep site choice was not influenced by rainfall on a short time scale, on a longer time scale, baboons chose to sleep closer to the area they had spent time in during the day, thus traveling less distance to their nightly sleep site. Another study found that while baboon rising times varied with visits from predators, the baboons did not move their sleep sites in response to predator interactions, nor did they change their sleep sites more frequently afterwards (Bidner et al. 2018). A study using accelerometers also found that baboons were more likely to experience lower sleep quality when sleeping in unfamiliar places, although time spent sleeping did not influence distance traveled the next day (Loftus et al. 2022). While these studies shed some light on the drivers of baboon sleep‐site choice, they were not able to account for, for example, seasonality limiting access to sleeping sites, or the drivers of choice in a low‐predator environment.

In this study, we use GPS data to examine the sleeping patterns of two troops of baboons in Gorongosa National Park, Mozambique (GNP). GNP is a unique study site to examine baboon behavior due to the relatively low density of predators at the time of the study, and the high abundance of baboons in the central portion of the park (Bouley et al. 2018; Gaynor et al. 2020; Stalmans et al. 2018). During the study, aerial counts showed 225 baboon troops in the central portion (1845 km^2^) of GNP, with troop size ranging from less than 10 individuals to over 200, compared to between 100 and 150 lions, around 50 wild dogs, and no resident leopards or hyenas, based both on predator monitoring data and aerial counts (Bouley et al. 2018; Gaynor et al. 2020; Hammond et al. 2022; Stalmans et al. 2018). Assuming an average troop size of around 90 individuals based on observation of troops on the floodplain and the studied troops, this indicates an average of around 11 baboons per square kilometer in Gorongosa, compared to 1.8 in KwaZulu‐Natal Province, South Africa (but note not all area surveyed was protected; Stone et al. 2012), and 1.3–12.1 in Cape Peninsula, South Africa (Hoffman and O'Riain 2012). However, given the lack of direct census data for individual baboons in GNP, it is difficult to make direct comparisons to other study sites.

Anecdotal reports from rangers in GNP also suggest that nocturnal movement is common, as are baboons sleeping on the ground. This could suggest that, due to the high baboon density relative to the low predator density in GNP during the time of the study, sleep sites may be harder to come by, and that conspecific avoidance may influence sleep site selection more than antipredation strategies for the studied population (Markham et al. 2013). Alternatively, the absence of nocturnal predators may allow for more nocturnal movement, which has previously been identified in diurnal primates, and less pressure to find a suitable sleep site by sunset (Ayers et al. 2020; Isbell et al. 2017).

In addition to the relatively low terrestrial‐predator‐to‐baboon ratio, with the exception of crocodiles, GNP is unique in being one of only two baboon study sites where a significant period of seasonal flooding occurs, the other being the Okavango Delta (Cheney and Seyfarth 2007). Annual rains result in a displacement of animals from the park's central floodplain, with terrestrial animals returning weeks later when the water has receded, as shown by detections in camera traps, satellite imagery of the flooding, and direct observation within the park (Beardmore‐Herd et al. 2025; Walker et al. 2023). While the timing and extent of this annual displacement vary, even in major events such as cyclones, baboon populations appear to be robust to seasonal changes (Böhme et al. 2006; Beardmore‐Herd et al. 2025).

Very few study sites combine the low predator risk and troop density seen in Gorongosa National Park during this study. This research aims to compare the baboons' sleeping patterns in this mosaic ecosystem with other sites where troops have been long studied, including sites with strong seasonality or high predation risk. We describe the sleeping sites in the two studied troops, the Woodland troop (WT) and the Floodplain troop (FT). We present data on the location and distances between sleep sites, how often and in what temporal patterns sleep sites are reused, the number of sleep sites per troop, and the variation in sleep sites between the wet and dry seasons, in order to establish a baseline for inter‐site comparisons and comparison within GNP over time. This study aims to answer three main questions: (1) Do baboons in GNP exhibit preferences for the location of sleep sites (e.g., center vs. periphery of their home range)? (2) What is the relationship between sleep site selection and areas where the baboons repeatedly spend time (areas of interest, or AOIs)? (3) Do the baboons return to the same sleep sites following seasonal displacement by flooding?

We begin by taking a basic measure of sleep site location, relative to the home range, to compare with other studies. Research at other baboon study sites has shown that baboons tend to sleep away from the peripheries of their home range, in presumably more familiar areas, and that these sleep sites are often used by multiple troops (Altmann 1974; Cheney and Seyfarth 2007; Loftus et al. 2022; Markham et al. 2016). This may be a product of avoiding inter‐group conflict in overlapping home ranges, an effect amplified in periods where sleep sites may be limited (Markham et al. 2013). With the density of baboons in GNP, a large amount of overlap was observed in the home ranges of both troops, even within central areas (Ferreira da Silva et al. 2025; Stalmans et al. 2018). Additionally, low predation risk and seasonal movement may expand the number of “familiar” areas available to both troops, but particularly FT, who undergo seasonal displacement. Therefore, asking this question allows us to establish whether the known behavior of sleeping centrally in the home range in other baboons exists in Gorongosa baboons, and whether this varies seasonally, when differences in troop density are expected.

We next test the hypothesis that there is a relationship between sleep site and AOIs. We begin by describing a technique to identify AOIs using cluster analysis of GPS points, identifying areas where the baboons spend more than 15 min multiple times over the course of a month. This technique creates a map of potential resource locations that would be otherwise unidentifiable due to the lack of observational data.

We then calculate the distance from the sleep site to both the last AOI of the previous day and the first AOI of the following day. A study on sleep site selection at the Amboseli Baboon Research Project found preliminary evidence that one factor of sleep site selection is proximity to a temporary or unexploited food resource (Markham et al. 2016). Another study on Anubis baboons ( *P. anubis* ) found that, over longer periods (i.e., a year), baboons may change their sleeping patterns based on climatic factors such as rainfall, which influences both the amount of food and water in the environment (Suire et al. 2021). Studies of other primate taxa have also shown a preference for sleeping close to resources, in particular food (Brividoro et al. 2019; Chapman 1989). Although we cannot know for certain the nature of the AOI the baboons are using, we presume they are some type of exploitable resource that can be used for food, water, shelter, or safety. As in other studies, we expect that the baboons will either travel a short distance from the last AOI of the day to the sleep site, or a short distance from the sleep site to the first AOI of the next day. However, we do not expect that the baboons will travel a long distance both from the last AOI and to the first AOI, instead conserving energy by minimizing travel distance between the sleeping site and an exploitable AOI.

Finally, we examine whether the baboons reuse sleep sites across seasonal displacement. While other troops use the same sleep sites repeatedly throughout the year (Bidner et al. 2018; Markham et al. 2016), GNP is unique in that some sleep sites are physically inaccessible to some troops due to seasonal flooding. Observations of FT and WT across 2017–2019 show that the baboons return to their dry‐season home range annually and spend time in similar areas, despite a period away from it. To test whether the baboons also reuse sleep sites across seasons, we calculate the number of days between sleep site uses. We expect to see baboons return to their dry season sleep sites after the wet season and return to sleep sites even after long periods of absence, due to familiarity with the area.

Although this study utilizes large quantities of GPS data, behavioral observations did not occur for the majority of the study. Therefore, by comparing AOIs identified by hierarchical clustering with known resource locations that were able to be ground‐truthed, and using the same method to identify AOIs that were not directly observed, we are able to test a method of analysis that may allow researchers more options for analyzing historic GPS data.

## 2 Methods

### 2.1 Study Subjects and Location

Data were collected in GNP. GNP is a 4000 km^2^ park located in central Mozambique. GNP is a highly mosaic, seasonal environment that experiences a distinct dry (April–October) and wet (November–March) season. Depending on the season, temperatures in open areas can reach into the high‐40s Celsius, while the nighttime lows reach around 5°C in the southern portion of the park. The seasonality of the area leads to the floodplain of GNP surrounding the central Lake Urema becoming inundated annually, as well as causing seasonal rivers to fill up and often burst their banks; during the dry season, the floods recede, leaving behind seasonal rivers that eventually dry into small ponds or empty riverbeds before the next dry season (Herrero et al. 2020; Stalmans and Beilfuss 2008). This pattern is further exacerbated by regular cyclones hitting the area, including Cyclone Idai 6 months prior to the study, which had long‐term impacts on the fauna of GNP, and led to an unusually lush dry season (Walker et al. 2023). During the Mozambique Civil War (1977–1992), the mammal population of GNP was decimated, leading to a near absence of predators in the park; however, the Gorongosa Restoration Project has since restored much of the herbivore population, as well as reintroduced and supported several predator species in GNP (Atkins et al. 2019; Bouley et al. 2018; Gaynor et al. 2020; Pansu et al. 2019). At the time of the study, GNP had a stable density of baboons, as well as a stable population of crocodiles and increasing numbers of lions and wild dogs (Stalmans et al. 2018).

Four chacma baboons ( *Papio ursinus griseipes* ), from two different troops, provided data. All four baboons were cycling, adult females with no dependent offspring, fitted with synchronous GPS/accelerometer collars between July and August 2019 and monitored for several months (see below for details). While relationship data between individuals within the group was unknown, baboons are female philopatric, and therefore collared females were expected to remain with the group through their entire study period. The two troops—the WT and FT—both reside in the southern portion of GNP. Both troops have been followed on foot and studied periodically since 2017 (WT) and 2018 (FT) as part of the Paleo‐Primate Project Gorongosa. Two of the collared baboons were from WT (Acacia and Kigalia), and two from FT (Eve and Abacaxi).

WT (*n* ~ 90 individuals) inhabits *Vachellia‐Combretum* woodland, maintaining the same central home range throughout the year, with the overall size of the home range shrinking approximately 15% through the wet season, based on analysis of the minimum convex polygon. This troop was observed briefly in 2017, then from April to November 2018, and again in July–August 2019. Observational data from this period indicate they subsist primarily on leaves, berries, leaf litter forage, and occasional bark stripping and hunting. The majority of their habitat is closed woodland that provides ample shade, and termite mounds that create cooler microclimates on hotter days, intersected by roads and two major pans. Because of the density of their habitat, their low habituation, and the size of the group, it is difficult to infer group mortality, relationships, and spatial distribution of individuals. The individuals were not all identifiable to the researchers.

FT (*n* ~ 30 individuals) ranges on the alluvial floodplain to the north and adjacent to WT's home range, an area comprised mostly of low grasses and sedges and edged with *Vachellia* trees. Because of the openness of their home range, there are limited available sleeping sites and very few shaded areas, and their daily ranges center around available water. Over the course of the day, the baboons tended to stop at the same areas of shade and water, using these sheltered areas to nap, socialize, and drink. They primarily eat sedges, underground corms, and seed pods, but have also been observed hunting baby antelopes, baby warthogs, snails, mussels, and small birds. FT's home range shifts during the wet season due to the inundation of the floodplain, forcing them to range in an area that they rarely travel to during the dry season. This troop was followed on foot from May to November 2018 and from July to November 2019. All adults were identifiable, and only one adult female disappeared during the course of the study. Most juveniles in the group, as well as some males, were not present in 2019 following Cyclone Idai, leading to a majority adult and subadult population during the study.

Observational data taken on these troops in 2018 and 2019 show that they prefer to sleep in trees, often cyclically returning to the same areas of trees. However, the exact sleeping site of each troop was unable to be identified in person due to constraints on working in GNP, which required a return to camp before the baboons had ascended their sleep trees. Nevertheless, baboons in both troops were often seen resting and socializing in the same areas both in the evenings and first thing in the morning, implying their sleep tree was nearby. However, within GNP, baboons were often seen on the roads or walking around after dark, and were observed by rangers to sleep on the ground, indicating that baboons were not always constrained by daylight or habitat to find a sleep site.

At the time of the study, lions and wild dogs ranged in both troops' home ranges, although neither predator was expected to hunt baboons overnight. No resident leopards, the primary nocturnal predator of baboons, were known to inhabit the area. Home range overlap was observed with multiple other troops, and intergroup encounters occurred weekly in FT, although there is no evidence that WT and FT ever encountered each other.

### 2.2 GPS Collars

Baboons were collared in July–August 2019 using cage trapping (*n* = 1) and free‐darting (*n* = 3). Each baboon was sedated and fitted with a GPS/accelerometer collar (model 1D, e‐obs Digital Telemetry, Grünwald, Germany) by an experienced GNP vet (see “Ethics Statement”). Collars were programmed to record a burst of GPS fixes every 15 min UTC time, and to collect accelerometer data at a rate of 20 Hz every minute, although 2/4 accelerometers failed soon after deployment. The collars then collected data through to the end of their battery life, which ranged from 293 to 323 days across the four baboons. Data were downloaded monthly from August 2019 to November 2019, then again in February 2020, May 2020, and June 2020, using an eObs BaseStation II connected to a Yagiantenna (868 MHz 10E) at a distance of 10–50 m and processed using eObs decoder_v10s1. Data downloads were conducted either by locating the troop using radio telemetry or by visual location, and attempts to download data persisted until all data was successfully downloaded. Within the FT, this generally took a single attempt, but took multiple attempts per download within the WT. Collars incorporated a fabric strap that would wear down after a minimum of 2 years, removing the collar from the baboon; therefore, collars were not designed to be retrieved.

### 2.3 Identification of Sleep Sites

Sleep sites were identified based on the baboons' location at 0200 h daily. In the event 0200 was not available due to collar failure, 0230 was used instead, as often the failure to connect to satellites persisted past the next attempted GPS fix. Due to variation in rising and sleeping times across the year and within the same troop, a single point was used to minimize error from averaging points across a set period of time. Once all sleep sites were identified, single‐linkage cluster analysis was used with a distance factor of 100 m to identify a single sleep site. Single‐linkage clustering, also known as “friends‐of‐friends” clustering, clusters groups from the bottom up. This means that points are initially clustered by the distance factor, with all points falling within that distance of each other becoming a single cluster. Following this, clusters, rather than points, that are within the distance factor combine into a single, larger cluster, regardless of whether all points within each cluster fall within the threshold. The distance factor of 100 m was chosen based on observation of tree clumps in the baboons' home ranges, as well as post hoc checking that no single sleep site could span an open area between two groves. This method also helped to account for choice in large patches of trees utilized by baboons for sleeping, wherein a number of trees used in a single patch of continuous woodland could be construed as a single sleep site for a large troop. Although this method may inadvertently combine distinct sleep sites, it allows for areas generally used for sleeping to be identified without ground‐truthing of the size, spread, and type of sleep sites themselves.

In both troops, the distance between points in a single sleep site cluster containing more than one point ranged from around 30 m to around 100 m. Because the nature of each sleep site was not known for analysis (e.g., single tree or group of trees), and the order of ascent into the sleep site was not observed, it was assumed that location within a troop's sleep site was not a choice that could be accounted for in analysis. Therefore, the geographic mean of the sleep site was used for analysis, given that this was generally less than 50 m from the actual location of the baboon.

Once sleep sites were identified based on GPS point clusters, the geographic mean of this cluster of points was then used as the sleep site location for analysis of the distance between sleep sites and between AOIs and sleep sites. Individual sleep sites, that is, the collared baboon location at 0200, were used for analysis of group spread.

Exact sleep sites were unable to be verified during this study, due to Park rules disallowing researchers from full dawn to dusk follows. However, sleep site GPS points were compared to satellite imagery and observational notes from 2018 and 2019 to identify types of sleep sites and the types of habitat they tended to be in. All identified sleep sites fell in forested areas, with trees of a suitable size for a baboon sleep site, based on measurements of preferred and non‐preferred sleep sites of baboons living in the Amboseli basin (Markham et al. 2016).

### 2.4 AOI Proximity

We identified non‐sleep site AOIs based on clustering analysis, using similar methods to previously described analysis of stop‐start trajectories (reviewed in Damiani et al. 2018). For each burst of points, the last GPS point was taken from the data to allow more time for the GPS to connect to the satellites for accuracy, particularly in densely wooded areas. GPS points were grouped by rolling 30‐day period, and filtered for points that only occurred after the baboon had already left the sleep site in the morning, or before they entered their evening sleep site. If the baboon spent more than 15 consecutive minutes (2 GPS fixes) within 30 m of the same spot, then those two fixes became a group of points (GoP). The 30 m cutoff was decided based on observational data taken for each troop, which approximately measured how far a baboon was likely to move within a food patch as opposed to moving to a new patch. Each GoP was added to until a fix was taken > 30 m from the original GPS fix of the GoP. Any GoP that had points within 40 m of a second GoP was combined with that GoP. A minimum convex polygon was then drawn around each final GoP, and this polygon was presumed to be an AOI of some type for the baboons, given that they stopped in that area for > 15 min multiple times over the course of a rolling 30‐day period. The last AOI of the day was defined as the final AOI the baboon utilized before entering the sleep site. The first AOI of the day was defined as the first AOI the baboon visited and remained in after exiting the sleep site. Distance to each AOI was calculated based on straight‐line Euclidean distance from the sleep site to the first utilized point of the AOI (i.e., first point stopped at in the GoP that makes up the AOI).

### 2.5 Distance to Home Range Edge

The centrality of a sleep site in the home range was categorized seasonally. First, GPS points that were obvious outliers (i.e., requiring movement of an impossible distance, usually > 10 km, from the normal ranging area of the baboons, within a 15‐min window) were removed.

Analysis of home range was undertaken in two ways. Because of the disparity between observational data for both troops, the robustness of complex home range analysis fully representing each troop was limited. To mitigate this, we analyzed home range size and edges in two different ways. The first was using the minimum convex polygon. Concentric minimum convex polygons were then drawn around the most central 5% of points, with each subsequent polygon adding another 5% of all points. The home range size of each polygon was plotted linearly. Visual examination showed a steep increase in home range size from 95% of points to 100% of points compared to every other increase, indicating that the least‐central 5% of points were outliers. These points were therefore excluded from the analysis of the home range boundaries, and the minimum convex polygon home range was drawn around the central 95% of points.

In addition to analyzing the minimum convex polygon, kernel density analysis was performed using the adehabitatHR package (Calenge 2006). Using the package's “kernelUD” function with *h* estimated using the argument “href,” all points for each troop were analyzed by season to determine a home range kernel. The vertices of his kernel were then extracted at 95%, and the edges of the resulting home range polygons were used as the edge of the home range.

Once the home range was calculated by season, the straight‐line distance from each day's sleep site to the closest home range edge for that season was calculated; this number was used regardless of whether the point fell inside or outside of the 95% centrality polygons.

### 2.6 Seasonality

For analysis, the year was divided into seasons based on calendar dates. The wet season was defined as November 1 to March 31, while the dry season was defined as April 1 to October 31 (Tinley 1977). The data used in this study were collected in the seasons following a major cyclone, which changed the dynamics of food and water availability by creating a particularly lush dry season (Walker et al. 2023). There were no major weather events during the study period besides the usual expected flooding, for which data is not currently available.

### 2.7 Group Cohesion

Given that each troop contained two collared baboons, who were presumed to have slept in proximity each night, the straight‐line distance between each troop's baboons' sleep site was calculated for each night both baboons had active collars (FT *n* = 293, WT *n* = 276). The average straight‐line distance and standard deviation were then calculated for the distance between baboons in each group, as an indicator of group spread or cohesion while sleeping. Because only two baboons are not necessarily representative of the group spread, these numbers serve as an estimate for the minimum spread of the group. Sleep sites were further compared to satellite imagery and observational data to compare tree patches used for sleeping within the same troop on the same night.

### 2.8 Ground‐Truthing Data

Both troops in this study were followed during habituation from May 2018 to November 2018. In addition, FT was observed on foot from July 2019 to November 2019; WT was observed sporadically in July and August 2019, daily in the weeks immediately following collaring, and then for a half day fortnightly until November 2019. During this time, habitat type, density, and vegetation height were noted, and scan samples of visible troop members were taken at 15‐ to 30‐min intervals, alongside focal behavioral sampling (Hammond et al. 2022). This data was used to verify habitat types and group spread, as well as to compare identified AOIs with known feeding sites.

### 2.9 Statistical Analysis

To test whether distance from the home range edge, distance to the first morning AOI or distance to the last evening AOI predicted sleep site choice, a linear mixed effects model was used from the R package “lme4” (Bates et al. 2015). The number of times each sleep site was used was predicted by distance to home range edge, distance to the first morning AOI, and distance to the last evening AOI as fixed effects, with season and individual identity as random effects. Data were analyzed by troop, with the same model run twice per troop: once using the minimum convex polygon home range, and once using the kernel density home range. *p* values and confidence intervals were determined using R package “stargazer” (Hlavac 2022), and residuals were tested for normality.

To further explore the relationship between the distance from the last AOI of the day and the first AOI of the next morning to the chosen sleep site, a paired Wilcoxon signed‐rank test was used, ranking the last AOI of each day with the first AOI of the following morning.

## 3 Results

### 3.1 GPS Data

GPS collars collected data from the moment of their placement until the day the battery died. All collars experienced some failure during data collection, and data were filtered to days where sleep sites were known (i.e., a GPS fix was acquired at either 2:00 or 2:30), and there were at least 8 h of GPS points throughout the following day. Collars failed to acquire, or failed to download, preprogrammed fixes < 1% of the time for all baboons except Acacia (WT), whose collar failed to acquire fixes or acquired an impossible fix around 4% of the time. For all baboons except Acacia, horizontal accuracy was measured as roughly 5 ± 5 m; for Acacia, accuracy was measured at 10 ± 8 m. Date of collar placement was removed from the analysis. This yielded 296 days of data for Eve (FT), 287 for Abacaxi (FT), 313 for Kigalia (WT), and 264 for Acacia (WT).

### 3.2 Within‐Group Sleeping Patterns

To analyze sleeping patterns, data for each group were filtered to only nights when sleep site data were available for both baboons within that group (FT *n* = 293; WT *n* = 276), regardless of whether GPS data for the day existed. Over 293 nights, the FT baboons were recorded to have slept on average 46 m apart (SD = 45 m; min = 2 m; max = 248 m). Over 276 nights, the WT baboons slept an average of 163 m apart (SD = 148 m; min = 2 m; max = 850 m). These maximum distances are considered the lowest maximum possible group spread, given that noncollared baboons may have slept farther apart than the collared baboons. However, even with group spread, comparison to satellite data showed that the collared baboons slept in continuous patches of woodland, rather than in separate patches. Baboons always slept in large groves of trees, as opposed to single trees in open areas, and slept more often in denser tree patches than in sparse tree patches. All baboons in FT were individually identifiable, and observational data indicated that subgrouping occurred rarely in the 2019 field season, and the troop was found as a cohesive unit each morning. However, similar data are not available for WT.

**FIGURE 1:**
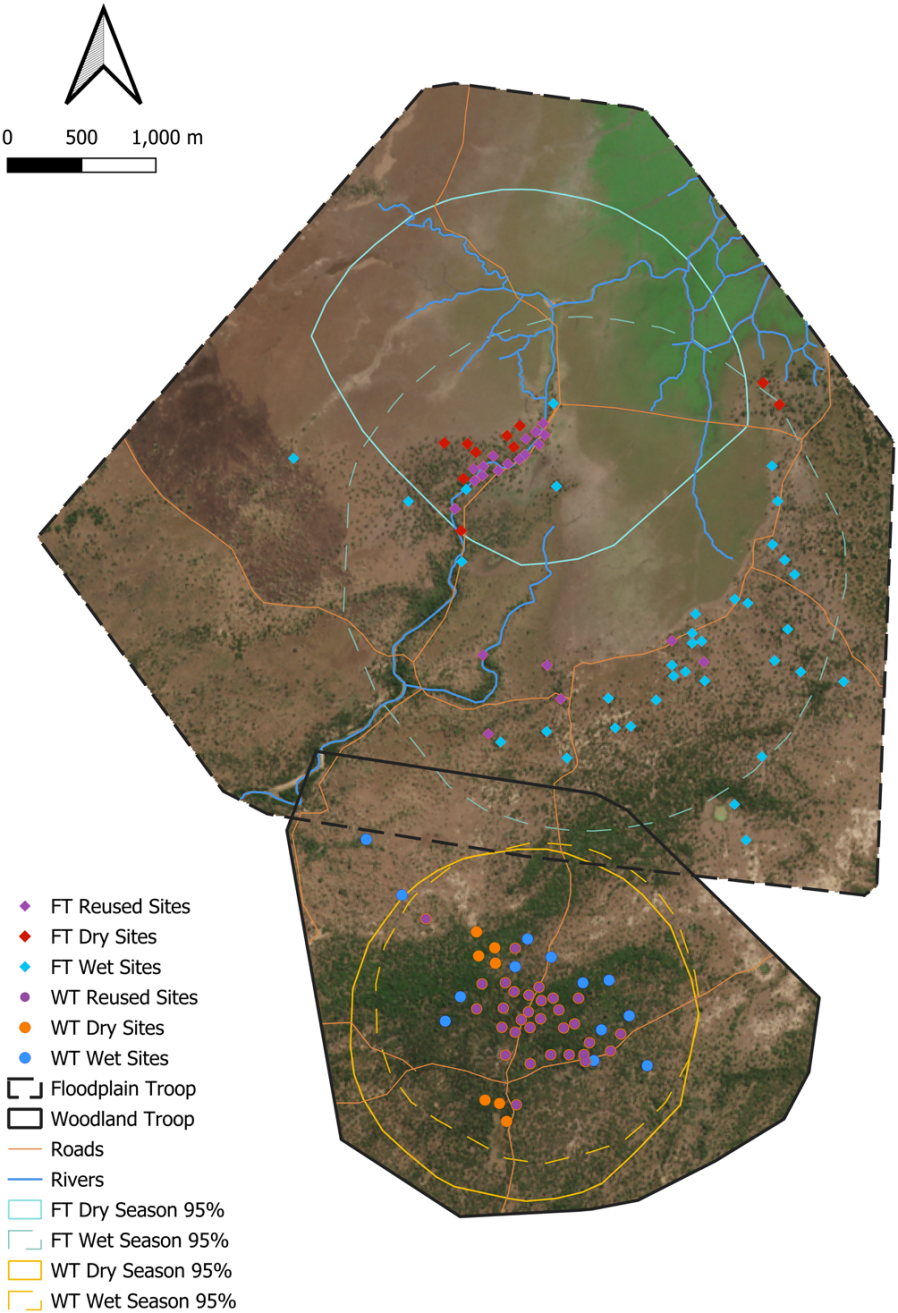
Annual sleep sites for study subjects. Colored points indicate whether sleep site was used in dry season (orange), wet season (blue), or reused across seasons (purple). Background is satellite imagery from October 2019, when there was a distinct contrast between large trees (primarily dark green) and open grassland areas (primarily brown, due to lack of rain). Roads and rivers are also marked. Home range boundaries represent the 95% minimum convex polygon of all GPS fixes seasonally, with dashed lines representing the wet season and solid lines representing the dry season. In total, FT slept in 10 sleep sites exclusively in the dry season, 35 in the wet season, and 21 in both seasons; WT slept exclusively in 7 dry season sites, 13 wet season sites, and 31 in both seasons.

In general, the baboons slept in more unique sleep sites in the relatively shorter wet season than in the dry season, although some sleep sites used in the wet season were also used in the dry season (Figure 1).

Baboons from FT showed a higher variation in sleep site use. Their most used sleep site alone accounted for 10% of their nights, while multiple identified sleep sites were only used once. Baboons from WT had more evenly distributed use of sleep sites, but still had multiple sites that were only used once (Figure 1).

To understand how each troop moved in their environment, movement from each collared baboon was combined, and the averages of both baboons were used. FT tended to move on average 400 m more between consecutive nights in the dry season compared to the wet season, while WT moved on average 200 m between consecutive nights across the whole year (Table 1).

**TABLE 1:** Summary of sleep site use for each baboon.

	Total nights	Unique SS (wet)	Unique SS (dry)	Avg Dist (wet) (*M* ± SD)	Avg Dist (dry) (*M* ± SD)
Eve (FT)	296	31	10	680 (±678)	207 (±526)
Abacaxi (FT)	287	31	11	653 (±692)	223 (±546)
Acacia (WT)	264	17	14	248 (±359)	223 (±305)
Kigalia (WT)	313	18	12	223 (±342)	217 (±297)

*Note:* The “Total Nights” column indicates how many nights for which sleep site data were known. The “Unique SS” columns indicate the total number of sleep sites used in each season. The “Avg Dist” columns show the average straight‐line distance between sleep sites on consecutive nights in each season.

### 3.3 Results of Linear Mixed‐Effects Model

We ran the same linear mixed‐effects model over the data from each troop. We found that for WT, distance from the home range edge significantly predicted the use of a sleep site, with sites further from the edge of the home range more likely to be used repeatedly. For FT, distance from the home range edge was similarly predictive. In addition, for FT, distance from the last AOI of the day to the sleep site was predictive of sleep site use, indicating that being closer to the last AOI used of the day is a significant predictor of sleep site use (Table 2). The same predictors were significant regardless of which method of home range delineation was used; however, when analyzed using the kernel density polygon, the home ranges of each troop were smaller, and therefore, the effect sizes grew for distance measurements.

**TABLE 2:** Results of linear mixed‐effects model predicting the number of times a sleep site was used, using distance to home range edge, distance to the last resource used before the sleep site, and distance to the first sleep site used after the sleep site as fixed effects.

	Woodland troop		Floodplain troop	
	MCP	Kernel density	MCP	Kernel density
Distance to home range edge	0.007*** (0.005, 0.009)	0.012*** (0.008, 0.017)	0.003*** (0.002, 0.004)	0.007** (0.003, 0.012)
Distance to last resource	−0.0002 (−0.001, 0.001)	−0.0001 (−0.002, 0.002)	−0.001** (−0.002, −0.0004)	−0.004*** (−0.006, −0.002)
Distance to first resource	−0.001 (−0.002, 0.00004)	0.0001 (−0.002, 0.002)	0.0002 (−0.0003, 0.001)	0.0002 (−0.001, 0.002)
Constant	−0.265 (−3.468, 2.937)	7.119*** (3.362, 10.976)	3.989* (0.619, 7.360)	12.495** (4.580, 20.410)
Observations	444	444	574	574
Log likelihood	−1158.586	−1508.602	−1589.350	−2147.53
Akaike Inf. Crit.	2331.171	3031.203	3192.699	4309.061
Bayesian Inf. Crit.	2359.842	3059.874	3223.168	4339.529

*Note:* All distances were straight‐line and measured in meters. Baboon ID and season were random effects. One model was run for both individuals in each troop. Model results display the effect estimate, with confidence interval in parentheses. The left column for each troop presents results of analysis using MCP home range estimation; the right column presents results of analysis using kernel density home range estimation.

* *p* < 0.05.

** *p* < 0.02.

*** *p* < 0.001.

### 3.4 Relationship Between Sleep Sites and AOIs

Of the 52 locations where at least one member of FT was recorded eating a non‐mammal food item between 2018 and 2019, 23 fell within identified AOIs. The majority of the remaining 29 were either food items that occurred in large patches that were generally eaten while moving, particularly the widespread *Cyperaceae* sedges (*n* = 21), or in areas that the baboons did not go to during the collared period but were identified previously (*n =* 6). In addition, some AOIs aligned with non‐consumable resources; for instance, patches of trees where the baboons frequently went to nap on hot days, or areas where they stopped to socialize and groom most evenings, as well as water sources (LLB, unpublished data). The baboons used the same AOI last night and returned to the same AOI first thing the next morning about 20% of the time. A Wilcoxon signed‐rank test comparing the distance to the first AOI of the day to the distance from the last AOI of the previous day was run for each troop, with combined data from both baboons. For both troops, it was found that the distance from the sleep site to the first AOI of the day was significantly longer than the distance from the last AOI of the previous day to the sleep site, with a moderate effect size (Table 3).

**TABLE 3:** Results of Wilcoxon signed‐rank tests comparing distance traveled from the sleep site to the first resource of the day to the distance traveled from the last resource of the previous day to the same sleep site.

	*V*	*p*	Effect size	*N*
Floodplain troop	113,654	< 0.01	0.33	574
Woodland troop	76,709	< 0.01	0.48	444

### 3.5 Return to Sleep Sites

When comparing sleep site use between the first and second dry seasons of the study, each baboon returned to more than half the sleep sites previously used before the wet season (Eve 14/22; Abacaxi 14/20; Kigalia 20/27; and Acacia 10/18). Additionally, each baboon returned to a previously used sleep site after an extended period of not using the sleep site; Abacaxi's longest absence from a sleep site was 171 days, Eve 275, Kigalia 277, and Acacia 207. Sleep sites that were returned to tended to be in the troop's core home range, although occasionally sleep sites on the periphery of the home ranges were used in both seasons. However, in both seasons, several sleep sites were used fewer than five times per baboon, while some sites were reused over 20 times.

## 4 Discussion

The sleeping patterns of primates have been long studied (Anderson 1984); however, few sites present such significant seasonal changes in rainfall combined with a low density of predators and a robust population of baboons. We found that, like in other baboon study sites, the baboons prefer to sleep in the more central part of their home range, and in the case of GNP, this pattern holds regardless of seasonality. However, because of the lack of wet‐season observational data, there is no definitive way of knowing when baboons are unable to access each part of their home range and when they choose not to. Ongoing analyses of camera trap data in the park (e.g., Beardmore‐Herd et al. 2025) show distinct changes in the spatial distribution of baboons following flooding, but little change in population, indicating that the baboons of the park are prepared to behaviorally adjust to survive flooding. However, studies focusing on the larger baboon distribution in GNP are limited. Flooding occurs in waves at the beginning and end of the wet season in GNP; therefore, areas can become accessible or inaccessible in a matter of days or hours. Further, aside from a 1‐ to 2‐month period of peak flooding, baboons in the dry season can physically access their wet season range and the sleep sites therein sporadically, depending on rainfall, and their choice to do so or not is informative of their sleep site choices. Unfortunately, complete flooding data is not available for this study period, which makes it difficult to fully separate seasonal data and untangle the differences between choosing a sleep site vs. available access to a sleep site. Future work should use flood monitoring data, as well as observational data, to understand the physical limitations of the baboons' ranges and sleep site choice.

In addition, despite lower predator density than other parks, we found that baboons moved sleep sites after just a few nights, as seen in other areas (Markham et al. 2016). We also found a large difference in the number of sleep sites used in the wet vs. the dry season. This implies that the baboons changed sleep sites more often in the wet season, and, for FT, moved farther between sleep sites. During the wet season, all mammals and other nonaquatic life are forced to move away from the floodplain to higher ground. This means that the animals are more condensed in a smaller area, which may increase the risk of predation and inter‐group encounters as seen in other study sites where seasonal flooding occurs, such as the Okavango Delta (Beardmore‐Herd et al. 2025; Cheney and Seyfarth 2007). As most of the sleep site reuse occurred in the dry season, it is possible that the baboons moved sleep sites more often in the wet season due to higher perceived predation risk from more frequent interactions with diurnal carnivores such as lions and wild dogs, and higher intergroup conflict. Additionally, a wetter environment combined with unpredictable flooding could prompt the baboons to move more often to avoid high parasite loads, low ground, or highly competitive areas. Because FT is forced to move into areas that are already the home ranges of established baboon troops due to the flooding, they may also be less familiar with where competition for sleep sites is high, the demography of other troops, or which sleep sites still exist from the previous year. During the wet season, FT monkeys slept within 200 m of the road surrounding the floodplain the majority of the time; they only spent four nights more than 500 m from this road, indicating that they are remaining on the edge of the flood water and may be more limited in which areas they can access. However, further data on the extent of flooding are needed to support this theory.

Conversely, WT would be expected to have their normal home range encroached upon by the higher density of mammals in the area, presumably increasing intergroup encounters and predator interactions as the spatial distribution of all mammals adjusted for the flooding. While they did not show such high preference for a single sleep site as FT, they also frequently moved sleep sites and had a number of sleep sites that were used less than three times over the study period. This may be due to perceived predation risk, or to remain temporally separate from other troops that overlap home ranges. In addition, WT was more limited by water during the study period, with their main water sources drying up before the onset of flooding in late 2019. This meant they were forced to walk long distances daily to the Mussicadzi River to drink, or to sleep by the river in an area they did not normally occupy, also increasing their risk of intergroup encounters as water became a limited resource. Further observational data and comparison of movement with water availability should be undertaken in future studies to understand the effects of water availability on sleep site choice.

Our results also showed that the FT baboons slept significantly closer to their last AOI of the day, regardless of how far they had to travel to their first AOI of the next day, a pattern that did not hold in WT. However, the Wilcoxon Signed‐Rank test indicated that there was a difference in distance between the last AOI of the day and the first AOI of the next day for both troops.

There could be several explanations for this. One might be that the baboons plan at least the last part of their day based on the sleep site they would prefer to end up at. In this case, the last AOI of the day might be planned or opportunistic, but most relevantly on the way to their sleep site. A second explanation could be predation avoidance. Lions and crocodiles are known predators of baboons, and are the predators in highest abundance in GNP, although to our knowledge, there were no successful predation attempts on the focal troops during the study (Cheney et al. 2004; Cheney and Seyfarth 2007). Since lions are known to hunt at dusk, baboons may be avoiding extra time spent on the ground during twilight, instead completing their evening socializing in the safety of their sleep site (Makin et al. 2017). This could further account for the difference in predictors between FT and WT. While WT has consistent access to shelter or trees to avoid predation attempts, FT must return to certain parts of their home range to have shelter at dusk. The areas that provide the most safety are also the areas with tall trees suitable for sleeping. Additionally, since many sleep sites are near bodies of water, baboons may return to the water to drink while there is still daylight, and have been observed resting and socializing by the water prior to moving to their sleep sites.

A third explanation is opportunism. Given the quantity of baboons in GNP and the lack of trees in certain areas, particularly in FT's home range, there should be some amount of competition for sleep sites. For troops in areas with low tree availability, many sleep sites may have already been claimed by troops. Therefore, it is safer to find a suitable sleep site earlier in the day and closer to the final AOI that may be suboptimal, but is available, rather than risk searching for a new sleep site that may have already been claimed and being left without one. This may imply that they chose AOIs close to the preferred sleep site, potentially to “stake their claim,” rather than a sleep site close to the final AOI.

Finally, it is possible that baboons are not planning sleep sites at all, but instead plan their last AOI of the day, and sleep where it is convenient at nightfall; this theory could account for the number of sleep sites used only once. Further analysis should consider ways to test these hypotheses individually, by incorporating behavioral data and monitoring intergroup or predator encounters when approaching or using each sleep site.

Our results further indicate that, even after an extended hiatus, baboons find and reuse sleep sites. Although the full period of displacement, where the most often used sleep sites are completely inaccessible for FT, lasts only for about 30–40 days of the wet season, we now have evidence to show that baboons not only return to the same areas after this absence, but also the same sleep sites as the year before. This is in line with other studies' findings that baboons are able to remember resources over at least the medium term (Noser and Byrne 2007, 2015). As severe weather events continue and the length of the rainy season changes, future studies should monitor how readily baboons return to previously inaccessible areas, as well as find familiar areas to reside during the flooding.

Overall, our study shows that the baboons of Gorongosa National Park's sleep sites are predicted by distance from home range edge, as well as proximity to the last AOI used each day for FT. This study forms a valuable baseline for baboon sleeping site decisions in GNP, which will undoubtedly change over time as more predators are introduced. Since the study, hyenas, leopards, and additional wild dogs have been released into the GNP. As the predator numbers grow, so will the risk to baboons, changing their habitat choices. The need to avoid predators that hunt in trees, such as leopards, may limit the number of available sleep sites for the baboons, due to the types of trees available. Further studies should look at the reuse of sleep sites in relation to the predation avoidance hypothesis, as well as the types of sleep sites used by baboons. Although the baboons have been seen to sleep in trees, reports of park baboons sleeping on the ground were common at the time of the study, and baboons were frequently observed walking around at night on the roads. Indeed, even our GPS data show some level of movement after dusk and before dawn, although rarely in the middle of the night (LLB, unpublished data). Preliminary analysis of rising and resting times in both troops shows variation in when baboons enter and leave their sleep sites between days even in the same season; the cause for this unfortunately cannot be verified without observational data. Future studies should look at the type of sleep sites selected, as well as utilize the accelerometer function of the collars of the troop to understand overnight movement and potentially act as a proxy for observational data. Additionally, other surrounding troops should be included in the data set to understand how competition for sleep sites plays into sleeping site decisions.

An additional limitation of this study was the method used to classify an AOI. Here, we used analysis similar to that used by mapping software to identify areas where individuals paused for a length of time (Damiani et al. 2018). Although many of the identified AOIs align with observational data of feeding sites, tracked by handheld GPS during observational follows, it is impossible to know the nature of all AOIs, as the studied baboons also stop frequently to nap, utilize shade, or groom in certain areas. Due to the limitations of traveling and observing baboon troops in GNP during the study, in part due to the wet season and in part due to the COVID‐19 pandemic, ground truthing of all AOIs could not occur. Without full understanding of what the baboons were doing at each AOI, our ability to interpret the results of this study is limited since the baboons may have different planning or motivation for different types of resources. Further testing of this methodology, alongside comparison to observational or accelerometer data, should be considered for future studies, either during observation or retrospectively comparing GPS data to observational data.

Finally, the inability to observe troops directly during the wet season means that validating the approach used in this study remains difficult. While GNP closes annually for the flood season, ongoing research will allow the timing and extent of flooding to be more closely monitored via remote sensing and data loggers. These data, combined retrospectively with GPS and accelerometer data, would allow for more robust conclusions about sleep site choice in the wet season.

This study presents the first data on sleeping site choice in the baboons of GNP. While it is limited in behavioral data, the number of GPS collars, and exact seasonal differences, it presents a potential way for historic GPS data to be analyzed to study sleep site choice. We found that despite differences in seasonality and predator density, baboons in this population exhibit similar patterns of sleep site choice as shown in other study populations with regard to distance from home range edge and AOI use, and establish a baseline of data for future studies looking at the sleeping patterns of baboons in GNP.

## Author Contributions

**Lynn Lewis‐Bevan:** conceptualization (lead), data curation (equal), formal analysis (lead), investigation (lead), writing – original draft (lead), writing – review and editing (equal). **Philippa Hammond:** data curation (equal), investigation (supporting), resources (equal), writing – review and editing (supporting). **Susana Carvalho:** conceptualization (supporting), methodology (supporting), resources (equal), supervision (equal), writing – review and editing (equal). **Dora Biro:** conceptualization (supporting), methodology (supporting), resources (equal), supervision (equal), writing – review and editing (equal).

## Ethics Statement

This work was carried out with ethical clearance from Oxford University (APA/1/5/ACER/23Jan2018) and from the Ministry of Tourism and the Gorongosa Restoration Project in Mozambique (permit numbers PNG/DSCi/C114/2018, PNG/DSCi/C93/2018, PNG/DSCi/C147/2019, and PNG/DSCi/C142/2019). All handling of the study subjects was performed by professional staff, and following collar deployment, all subsequent data collection was completed remotely and in the animals' natural habitat.

## Conflicts of Interest

The authors declare no conflicts of interest.

## Data Availability

The data that support the findings of this study are available from the corresponding author upon reasonable request.
